# Prediction of Grade Reclassification of Prostate Cancer Patients on Active Surveillance through the Combination of a Three-miRNA Signature and Selected Clinical Variables

**DOI:** 10.3390/cancers13102433

**Published:** 2021-05-18

**Authors:** Paolo Gandellini, Chiara Maura Ciniselli, Tiziana Rancati, Cristina Marenghi, Valentina Doldi, Rihan El Bezawy, Mara Lecchi, Melanie Claps, Mario Catanzaro, Barbara Avuzzi, Elisa Campi, Maurizio Colecchia, Fabio Badenchini, Paolo Verderio, Riccardo Valdagni, Nadia Zaffaroni

**Affiliations:** 1 Department of Biosciences, University of Milan, 20133 Milan, Italy; 2 Bioinformatics and Biostatistics Unit, Department of Applied Research and Technological Development, Fondazione IRCSS Istituto Nazionale dei Tumori, 20133 Milan, Italy; Chiara.ciniselli@istitutotumori.mi.it (C.M.C.); Mara.lecchi@istitutotumori.mi.it (M.L.); Paolo.verderio@istitutotumori.mi.it (P.V.); 3 Prostate Cancer Program, Fondazione IRCCS Istituto Nazionale dei Tumori, 20133 Milan, Italy; Tiziana.rancati@istitutotumori.mi.it (T.R.); Cristina.marenghi@istitutotumori.mi.it (C.M.); Fabio.badenchini@istitutotumori.mi.it (F.B.); Riccardo.valdagni@istitutotumori.mi.it (R.V.); 4 Molecular Pharmacology Unit, Department of Applied Research and Technological Development, Fondazione IRCCS Istituto Nazionale dei Tumori, 20133 Milan, Italy; Valentina.doldi@istitutotumori.mi.it (V.D.); Rihan.elbezawy@istitutotumori.mi.it (R.E.B.); 5 Department of Medical Oncology, Fondazione IRCCS Istituto Nazionale dei Tumori, 20133 Milan, Italy; Melanie.claps@istitutotumori.mi.it; 6 Division of Urology, Fondazione IRCCS Istituto Nazionale dei Tumori, 20133 Milan, Italy; Mario.catanzaro@istitutotumori.mi.it; 7 Division of Radiation Oncology 1, Fondazione IRCCS Istituto Nazionale dei Tumori, 20133 Milan, Italy; Barbara.avuzzi@istitutotumori.mi.it; 8 Department of Pathology and Laboratory Medicine, Fondazione IRCCS Istituto Nazionale dei Tumori, 20133 Milan, Italy; Elisa.campi@istitutotumori.mi.it (E.C.); Maurizio.colecchia@istitutotumori.mi.it (M.C.); 9 Department of Oncology and Hemato-Oncology, University of Milan, 20133 Milan, Italy

**Keywords:** prostate cancer, active surveillance, microRNA, biomarker

## Abstract

**Simple Summary:**

Active surveillance (AS) has evolved as an alternative to radical treatment for potentially indolent prostate cancer. However, current selection criteria for entering AS are suboptimal, and a significant percentage of patients discontinue AS because of disease reclassification. Hence, there is an unmet need for novel biomarkers for the accurate identification of high-risk PCa and the unequivocal classification of indolent disease. Circulating biomarkers, including microRNAs identified through liquid biopsies, represent a valuable approach to improve on currently available clinicopathological risk-stratification tools. In an attempt to identify specific microRNA signatures as potential circulating biomarkers, the authors performed an unprecedented analysis of the global microRNA profile in plasma samples from AS patients and identified and validated a three-microRNA signature able to predict patient reclassification. The addition of the three-microRNA signature was able to improve the performance of currently available clinicopathological variables, thus showing potential for the refinement of AS patients’ selection.

**Abstract:**

Active surveillance (AS) has evolved as a strategy alternative to radical treatments for very low risk and low-risk prostate cancer (PCa). However, current criteria for selecting AS patients are still suboptimal. Here, we performed an unprecedented analysis of the circulating miRNome to investigate whether specific miRNAs associated with disease reclassification can provide risk refinement to standard clinicopathological features for improving patient selection. The global miRNA expression profiles were assessed in plasma samples prospectively collected at baseline from 386 patients on AS included in three independent mono-institutional cohorts (training, testing and validation sets). A three-miRNA signature (*miR-511-5p*, *miR-598-3p* and *miR-199a-5p*) was found to predict reclassification in all patient cohorts (training set: AUC 0.74, 95% CI 0.60–0.87, testing set: AUC 0.65, 95% CI 0.51–0.80, validation set: AUC 0.68, 95% CI 0.56–0.80). Importantly, the addition of the three-miRNA signature improved the performance of the clinical model including clinicopathological variables only (AUC 0.70, 95% CI 0.61–0.78 vs. 0.76, 95% CI 0.68–0.84). Overall, we trained, tested and validated a three-miRNA signature which, combined with selected clinicopathological variables, may represent a promising biomarker to improve on currently available clinicopathological risk stratification tools for a better selection of truly indolent PCa patients suitable for AS.

## 1. Introduction

Prostate cancer (PCa) is the third most common malignancy and the fifth cause of cancer death in men worldwide [1], with the number of diagnoses markedly increased after the introduction of the prostate-specific antigen (PSA) screening. Although PSA screening has contributed to the reduction of PCa-related mortality [2], the limited specificity for detecting clinically significant disease has resulted in over diagnosis of many clinically insignificant cancers, in turn leading to overtreatment of patients and their exposure to the treatment side-effects, without meaningful benefit.

Active surveillance (AS) has emerged as an alternative strategy in selected men with favorable-risk disease [3]. It consists of a strict monitoring protocol aimed at avoiding treatment unless there is evidence of disease reclassification, thus delaying radical treatments without losing the window of curability. Though patient selection criteria can slightly vary between different AS programs, they are mainly based on conventional clinical and histopathological criteria, including Gleason pattern score (GPS), clinical T-stage, PSA, number of positive cores at biopsy and PSA density [4]. In AS cohorts, conventional triggers for intervention are PSA kinetics (based on PSA doubling time or PSA velocity), clinical progression, upsizing (increase in the number of positive cores on repeated biopsies or tumor extension exceeding protocol cut-offs) and upgrading (increase in GPS on a repeated biopsy) [4]. In recent years, upgrading has been widely considered the most relevant and reliable criterium for AS discontinuation.

The efficacy and safety of AS have been assessed in several long-term prospective AS cohorts, showing a 15-year cancer-specific mortality ranging from 0.2 to 5% [5]. However, the main challenge of AS lies in accurately selecting patients to properly identify those with truly low-risk PCa and exclude those who harbor occult high-risk cancer foci.

At present, about 20% of patients discontinue AS due to upgrading to higher risk disease at the time of the first repeated biopsy [5], highlighting the necessity to improve the accuracy of selection criteria in distinguishing true pathology of PCa upfront. Based on this premise, there is an unmet need for novel biomarkers to reduce misclassification. To this purpose, multiparametric magnetic resonance imaging (mpMRI) has been recently implemented into AS protocols to ameliorate tumor sampling and improve risk estimation [6].

Emphasis has also been placed on developing minimally invasive tests aimed at assessing the presence of biological markers in blood and urine for refining the disease state. Although these biomarkers have not been robustly studied in AS cohorts, encouraging results have been reported for some of them. For instance, the serum 4Kscore, which evaluates a panel of four kallikrein proteins (total PSA, free PSA, intact PSA and human kallikrein-related peptidase 2) combined with clinical parameters, was found to improve the accuracy of predicting reclassification at the first surveillance biopsy in the multi-institutional Canary Prostate Active Surveillance Study (PASS) [7]. Another serum PSA-based biomarker, the prostate health index (PHI), a mathematical formula consisting of total PSA, free PSA and [−2]proPSA isoform of PSA, was found to be predictive of grade reclassification in the Johns Hopkins AS cohort [8]. Moreover, urinary prostate cancer antigen 3 (*PCA3*) proved to be associated with reclassification at the first repeated biopsy in the PASS cohort [9].

MicroRNAs (miRNAs) are endogenous small non-coding RNA molecules regulating gene expression mainly at the post-transcriptional level [10]. In the last 15 years, a great effort has been made to investigate the role of miRNAs in human cancer, and plenty of publications have shown their aberrant expression in almost all tumor types as well as a direct functional involvement of many of them in carcinogenesis [11]. More recently, interest has been devoted to the validation of miRNAs as potential circulating biomarkers [12]. In PCa, a number of studies have attempted to identify circulating miRNAs, the levels of which may discriminate between patients and healthy individuals or correlate with disease status, stage, aggressiveness and response to therapy, as reviewed in [13].

Here, through the assessment of global miRNA expression profile in plasma samples from PCa patients at inclusion in AS, we trained, tested and validated a three-miRNA signature able to (*i*) predict upgrading at the first repeated biopsy in three independent patient cohorts and (*ii*) improve on the available clinicopathological risk stratification criteria for patient enrollment in AS.

## 2. Materials and Methods

### 2.1. Study Cohorts

Low-risk PCa patients included in the study were prospectively accrued into AS protocols open to enrollment between 2009 and 2018 at Fondazione IRCCS Istituto Nazionale dei Tumori di Milano (INT) [14]. Details of the inclusion criteria, follow-up schedules and indications for AS discontinuation are summarized in Table S1.

Plasma samples were collected at patient inclusion in AS programs. All patients provided written informed consent to donate blood samples for research purposes. The first repeated biopsy was planned at 1 year from diagnosis. Original grading of study biopsies was assigned using the ISUP 2005 guidelines. A recent revision by the study pathologist (M. Colecchia) showed that all GPS = 3 + 3 cores can be attributed to new prognostic grade group 1 (PGG1), according to the Consensus Conference ISUP 2014 criteria.

On the basis of biopsy histopathological results, we categorized patients into indolent or upgrading populations, the former including individuals still fulfilling all protocol inclusion criteria and the latter comprising patients found to bear GPS > 3 + 3/≥ PGG2 tumor cores and who had dropped out from AS. Patients who had dropped out for other reasons were not included.

**Figure 1:**
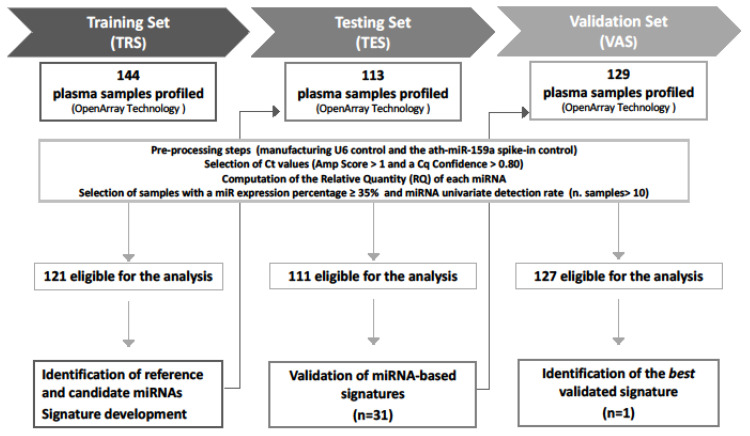
Study workflow. A total of 386 plasma samples were sequentially collected from low-risk PCa patients at the inclusion in AS protocols. The global miRNA expression profiles were assessed by OpenArray technology in all sample sets. Generated data went through preprocessing steps to define samples eligible for the analysis. Training set (TRS) data were analyzed to identify (i) reference miRNAs for normalization and (ii) candidate miRNAs associated with disease reclassification (univariate analysis) and (iii) to develop miRNA signatures. Thirty-one miRNA signatures were then validated in the testing set (TES), and the best signature retaining statistical significance in the validation set (VAS) was identified.

The study comprises three independent prospectively collected patients’ cohorts: (i) a training set (TRS), including 144 patients (121 indolent and 23 upgrading) collected between 2009 and 2013; (ii) a testing set (TES), including 113 patients (93 indolent and 20 upgrading) collected between 2010 and 2016; and (iii) a Validation Set (VAS), including 129 patients (106 indolent and 23 upgrading) collected between 2012 and 2018. Figure 1 reports the design of the study, which was approved by the Institutional Ethical Committee of INT (project approval code: INT 10/11).

### 2.2. Plasma Preparation, RNA Extraction and miRNA Profiling

Plasma was processed immediately after blood withdrawal using a double centrifugation protocol developed to minimize hemolysis and allow proper miRNA detection [15]. Only frankly hemolyzed samples were discarded. Plasma samples were stored at −80 °C until use.

RNA was extracted from 200 μLof plasma using the MiRNeasy kit (Qiagen, Hilden, Germany) per manufacturer’s protocols with minor modifications and bacteriophage MS2 RNA as carrier. *Ath-miR-159a* spike-in RNA (6.4 × 10^8^ copies) was added before RNA extraction. RNA was then quantified by Qubit and quality checked on TapeStation4200 (Agilent, Santa Clara, CA, USA).

Circulating miRNA expression was measured using the PCR-based TaqMan OpenArray (OA) Human MicroRNA Panel Technology (Thermo Fisher Scientific, Waltham, MA, USA), which assesses 754 miRNAs and 4 control RNAs in replicates. RNA was reverse-transcribed and cDNA mixed with 2.5μLof OA real-time PCR master mix. Then, the reaction mixture was dispensed onto the OA plate using an automated sample loading system, AccuFill (Thermo Fisher Scientific, Waltham, MA, USA). Samples were allocated on the OA plates based on an ad hoc randomized list according to both the disease state (indolent vs. upgrading) and the year of collection. Real-time PCR was performed using Quantum Studio 12K Flex (Thermo Fisher Scientific, Waltham, MA, USA) and manufacturer’s proprietary software. After image acquisition, the threshold cycle (Ct) and the confidence parameter reflecting the quality of the amplification curve generated during PCR were calculated by Thermo Fisher Scientific’s proprietary algorithms and used for further data analysis.

Finally, to assess the influence of hemolysis contamination on miRNA expression, an ad hoc hemolysis forced experiment (HFE) was implemented. Hemolysis was artificially introduced into the plasma sample from an indolent patient of the TRS by adding red blood cells starting from a known concentration and by performing three serial 1:4 dilutions (range, 0.004–0.25% *v*/*v*), for a total of five samples including the uncontaminated plasma. Each sample was analyzed in triplicate for the complete spectrum of absorbance on a NanoDrop 1000 (Thermo Fisher Scientific, Waltham, MA, USA) before RNA extraction. Samples were then profiled for miRNA expression by OA and analyzed according to the simultaneous confidence intervals approach [15].

### 2.3. Data Processing

Within each set of data (i.e., TRS, TES, VAS), the following preprocessing steps were applied: (*i*) selection of samples for which both the manufacturing *U6* control and the *ath-miR-159a* spike-in control were detectable; (*ii*) selection of Ct values with an Amp score >1 and a cycle of quantification (Cq) confidence >0.80; (*iii*) computation of the relative quantity (RQ) of each miRNA using the comparative Ct method following the formula 2^−^^ΔCt^, with ΔCt = Ct_miRNA[i]_ − Ct_reference_ [16]. For data normalization, we used the reference miRNAs identified by running an updated version of the normalization quantitative polymerase chain reaction array algorithm (NqA) [17]. TRS data were analyzed in a univariate manner to identify those miRNAs statistically associated with upgrading. In this selection step, only miRNAs detected in at least 10 upgrading and 10 indolent patients were considered. Candidate hemolysis-free miRNAs (according to the HFE) showing a significant association with upgrading in univariate fashion were then evaluated in multivariate fashion to build miRNA signatures [18]. The predictive capability of the identified signatures was evaluated on the TES and finally assessed on the VAS.

### 2.4. Statistical Analysis

In the TRS, the association between miRNA levels, on logarithmic scale log_2_ (RQ), and upgrading was assessed by resorting to the nonparametric Kruskal–Wallis test and to the logistic regression model. miRNAs showing at least one instance of significance in univariate fashion were then combined in multivariate fashion (i.e., all subset analysis). According to the required number of events per variable (EPV) [18], standard estimations or the penalized maximum likelihood estimation (PMLE) approach [19] was used. For each model, the predictive capability was calculated as the area under the receiver operating characteristic ROC curve (AUC) and its corresponding 95% confidence interval (95% CI). Signatures showing a statistically significant performance (in terms of predictive capability) in the TRS were then evaluated on the TES and, if confirmed, also on the VAS. Specifically, the statistical significance of the signatures was evaluated on the TES by applying the same regression coefficients obtained in the TRS, as reported in [20]. The best signatures were finally selected as those retaining their significance in the VAS (i.e., miRNA model). Furthermore, by considering the three cohorts all together, the role of each clinicopathological variable was evaluated. Firstly, the pairwise relationships between each of the considered clinicopathological variables were properly investigated according to their nature. Specifically, the association of each continuous variable with the categorical ones was evaluated by resorting to the nonparametric Kruskal–Wallis test; for comparing two categorical variables, a chi-squared test was applied. The strength of the association between continuous variables was assessed by Spearman correlation coefficient (r_S_), and its 95% CI was computed according to the bias-corrected and accelerated bootstrap method (95% CI_BCa_) [21]; only significant correlation reflecting a “good to excellent relationship” (i.e., r_S_ ≥ 0.75) was considered as relevant [22]. Variables showing a significant association with upgrading were jointly considered (i.e., clinicopathological model) with the miRNA model (i.e., full model) in a multivariate logistic regression model [23]. The nonparametric approach of De Long and Clarke-Pearson [24] was used to compare the discriminatory performance of different models. For each model, the predicted measures (linear predictor and corresponding estimated probability of upgrading) were also computed.

All statistical analyses were carried out with the SAS (version 9.4.; SAS Institute Inc., Cary, NC, USA) and R software by adopting a significance alpha level of 5%.

## 3. Results

Circulating miRNA profiles were assessed in plasma samples prospectively collected from 386 PCa patients at inclusion in AS programs at INT from 2009 to 2018. Table 1 reports the main demographic and clinicopathological characteristics of patients in the three cohorts included in the study. No appreciable differences were observed among the cohorts, with the only exception of positive core variables in the TRS/TES compared to the VAS.

**Figure 2:**
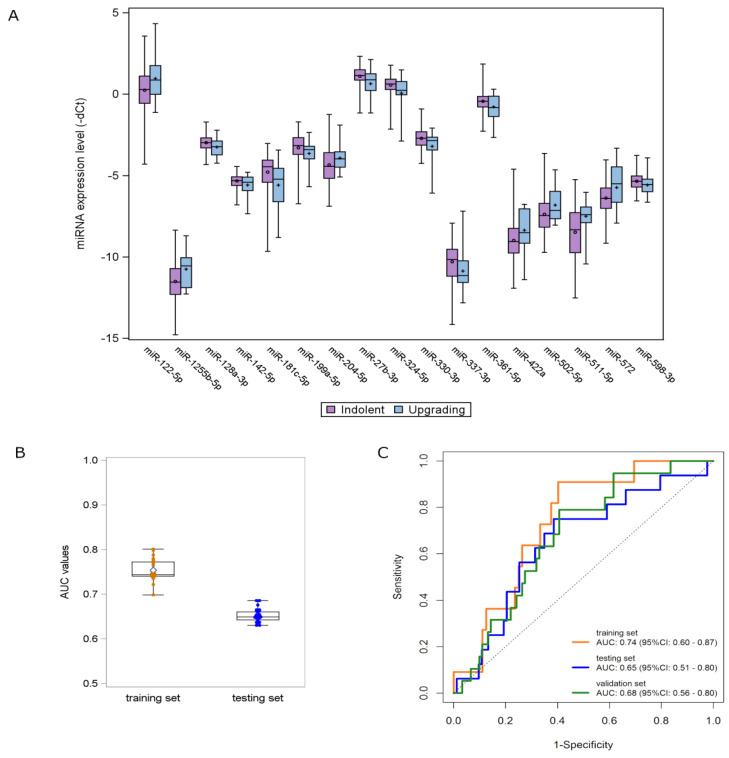
miRNA signature development and validation. (**A**) Distribution of the expression levels of the 17 deregulated miRNAs according to the upgrading (light blue) and indolent (purple) status found in the TRS. Each box indicates the 25th and 75th percentiles. The horizontal line inside the box indicates the median, and whiskers indicate the extreme measured values. (**B**) Box plots showing the distribution of AUC values of the 31 signatures identified in the TRS and validated in the TES. Each box indicates the 25th and 75th percentiles. The horizontal line inside the box indicates the median, and whiskers indicate the extreme measured values. (**C**) ROC curves of the 3-miRNA signature in the three AS patient cohorts: TRS (orange line), TES (blue line) and VAS (green line).

Using the updated version of the NqA [17], four miRNAs (*let-7c-5p*, *miR-126-3p*, *miR-26b-5p* and *miR-379-5p*) were selected to be used for data normalization to control intersample variability in the TRS cohort. A list of 17 miRNAs differentially expressed in plasma samples from upgrading compared to indolent patients was identified by univariate analysis (Table 2, Figure S1). Among candidate miRNAs, 7 were upregulated and 10 were downregulated in upgrading relative to indolent patients (Figure 2A).

By opportunely combining the candidate miRNAs in a multivariate fashion, 31 promising signatures, with a statistically significant performance and a high multivariate detection rate (i.e., miRNAs were detectable in >80% of upgrading samples), were identified in the TRS and validated in the TES by applying the same regression coefficients (Figure 2B). Among these signatures, a three-miRNA signature—consisting of *miR-511-5p*, *miR-598-3p* and *miR-199a-5p*—retained the statistical significance in terms of AUC also when tested in the VAS. The miRNA score was calculated for individual patients by weighting the expression of each miRNA as follows: (0.2700 × expression of *miR-511-5p*) + (−0.5848 × expression of *miR-598-3p*) + (−0.3441 × expression of *miR-199a-5p*) + (−4.020). The ROC curves of the miRNA model are depicted in Figure 2C. The AUC values range from 0.74 (95% CI: 0.60–0.87) in the TRS to 0.65 (95% CI: 0.51–0.80) and 0.68 (95% CI: 0.56–0.80) in the TES and VAS cohorts, respectively.

The performance of the available clinicopathological variables (age, volume, PSA density, number of positive cores at diagnosis, percentage of positive cores, maximum percentage of tumor in core biopsies) was then evaluated in the overall case series. As reported in Table 3, all the considered clinicopathological variables showed a significant association with upgrading in univariate analysis. By taking into account the results from the pairwise comparisons (Table S2), a multivariate clinicopathological model including age, PSA density and maximum percentage of tumor in core biopsies was fitted (Table 3). Notably, no statistically significant correlation was observed between the expression of the three miRNAs and clinicopathological variables.

**Figure 3:**
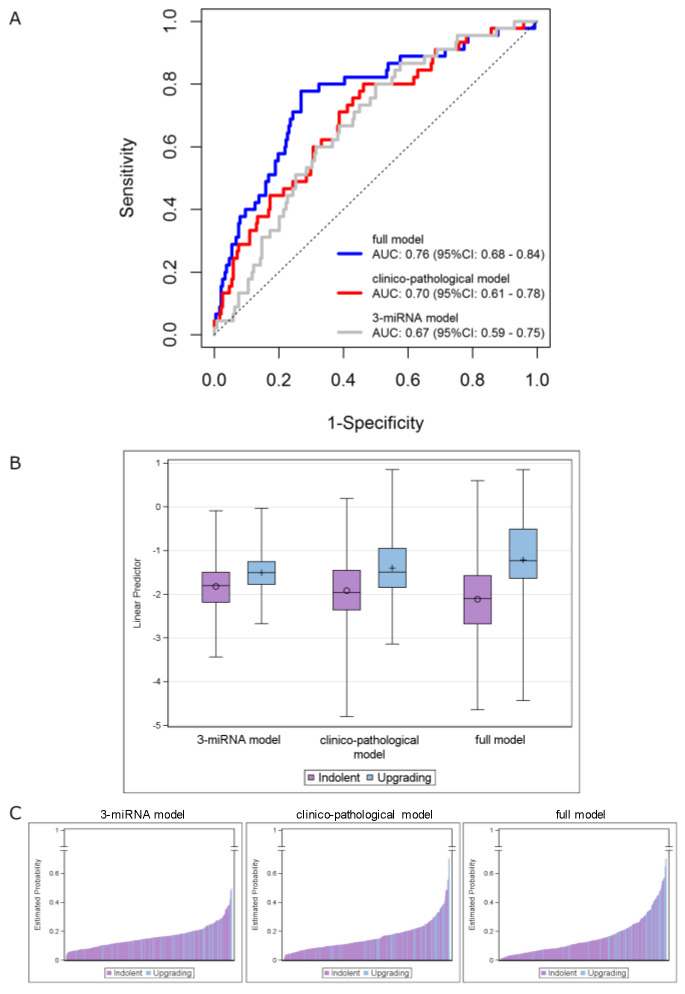
Integrating the 3-miRNA model into the clinicopathological model (including age, PSA density and maximum percentage of tumor). (**A**) ROC curves in the overall series of AS patients. ROC curves from 3-miRNA (gray line), clinicopathological (red line) and full (blue line) models. (**B**) Distribution of the linear predictors estimated for the 3 fitted models according to the upgrading (light blue) and indolent (purple) status, in the overall series. Each box indicates the 25th and 75th percentiles. The horizontal line inside the box indicates the median, and whiskers indicate the extreme measured values. (**C**) Bar plots showing the estimated probabilities for each patient according to the upgrading (light blue) and indolent (purple) status for the 3 fitted models (from left to right: 3-miRNA, clinicopathological and full model). The full-model score was calculated for individual patients as follows: (0.048 × age at biopsy) + (0.022 × Max PCa length) + (1.402 × PSA density) + (1.013 × miRNAscore) + (−0.344).

The performance of theclinicopathological model (AUC = 0.70, 95% CI: 0.61–0.78) was not substantially different (*p* = 0.658) from that of the three-miRNA model in the overall cohort (AUC = 0.67, 95% CI: 0.59–0.75). However, its performance was significantly improved (*p* = 0.007) when the miRNA score was included (i.e., full model), with the AUC value increasing to 0.76 (95% CI: 0.68–0.84) (Figure 3A). The distributions of the predicted measures of the fitted models according to the indolent/upgrading status are depicted in Figure 3B,C. As reported in Figure 3B, the linear predictors of upgrading samples show higher AUC values compared to the indolent ones, by moving from the three-miRNA model to the full model. Specifically, an improvement of the discriminatory capability was achieved by jointly considering the miRNAs and the clinicopathological data. The same observations can be drawn from Figure 3C, which reports bar plots of the estimated probabilities for each patient according to his status: higher estimated probabilities were obtained for upgrading patients when the full model was applied.

Figure S2 displays the corresponding results obtained by including all the available variables in the clinicopathological model. Of note, superimposable results were observed with respect to the more parsimonious model.

For the explorative purpose to illustrate the potential usefulness of the signatures, we pursued a further analysis by estimating the performance indexes after dichotomization of the linear predictor according to the Youden index based cutoff values. We obtained 80% (95% CI: 65–90%) sensitivity, 50% (95% CI: 43–57%) specificity, 23% (95% CI: 19–43%) positive predictive value (PPV) and 93% (95% CI: 86–95%) negative predictive value (NPV) for the three-miRNA signature. An appreciable increment was observed for specificity (73%, 95% CI: 67–79%), PPV (35%, 95% CI: 29–55%) and NPV (95%, 95% CI: 89–96%) by jointly considering the clinicopathological variables with the miRNA-signature, while a similar sensitivity value was retained (78%, 95% CI: 63–89%).

## 4. Discussion

In this study, we performed an unprecedented analysis of the circulating miRNome in plasma samples from three independent cohorts of low-risk PCa patients followed in AS protocols at INT in the search for biomarkers to improve the prediction of disease reclassification. We trained, tested and validated a three-miRNA (*miR-511-5p*, *miR-598-3p* and *miR-199a-5p*) signature associated with disease upgrading, which was shown to provide risk refinement to standard clinicopathological features.

The only previous study looking at blood miRNAs in the AS setting pursued a targeted approach by analyzing the expression of nine miRNAs, *a priori* selected based on the results of studies conducted on PCa patients and healthy individuals, in serum samples from two independent AS patients’ cohorts [25]. It was found that a three-miRNA (*miR-223*, *miR-24* and *miR-375*) score significantly predicted reclassification (defined as upgrading or PSA doubling time <3 years or MRI progression) and also conferred additive predictive value to PSA [25]. In a further study carried out on urine samples from an AS patient cohort, the same research group [26] reported that a three-marker panel, consisting of *miR-24*, *miR-30c* and *CRIP3* gene methylation, significantly predicted patient reclassification. Another report investigated circulating miRNAs in PCa patients potentially eligible for AS but who underwent radical prostatectomy [27]. The authors reported that presurgical serum levels of *miR-19*, *miR-345* and *miR-519c-5p* could help identify patients showing upgrading of the disease in the surgical specimen compared to the initial biopsy [28].

Circulating miRNAs are promising biomarkers in oncology but have not yet been implemented in the clinical practice given the lack of concordance across the studies, as can be easily inferred by the variable results obtained in AS or AS-eligible patients, which failed to identify common miRNAs. In this context, in our AS cohorts, we were not able to find any significant correlation between *miR-223*, *miR-24* and *miR-375* [25]—neither as individual miRNAs nor as three-miRNA signature—and patient reclassification. Methodological heterogeneity affecting several steps of circulating miRNA profiling (including sample source and preparation, profiling and data normalization) could represent a limiting factor for the identification and validation of reliable circulating signatures. In this regard, in the present study, we tried to control intersample variability by defining proper endogenous controls and also ascertained that our candidate miRNAs were not influenced by the presence of hemolysis, which is an important confounding factor in circulating miRNA analysis. In addition, penalized statistical approaches, according to the number of events pervariable (EPV) [19], have been implemented to account for the low number of upgrading patients.

Thus far, a relevant fraction of patients enrolled in AS drop out of the protocol due to upgrading at the time of a repeated biopsy [5]. Though progression from low (GPS = 3 + 3/PGG1) to intermediate or high grade (GPS > 3 + 3/≥PGG2) PCa cannot be completely excluded [29], a widespread view is that upgrading at confirmatory biopsy more likely reflects incomplete sampling on the diagnostic biopsy. In this regard, we recently showed that GPS = 3 + 3/PGG1 bioptic cores from upgrading patients may already harbor genomic lesions typical of more aggressive cancers, such as *PTEN* deletion or *MYC* amplification [28].

Based on the assumption that circulating miRNAs carry information coming from all tumor foci present in the prostate gland, they could complement the intrinsically limited information obtained through bioptic sampling. Ideally, they could be suitable for repeated measurements, in addition to or in place of repeated biopsies, to monitor disease status during AS. Indeed, in our study, we found that the three-miRNA signature on plasma samples collected at the baseline is significantly associated with upgrading at the first repeated biopsy. However, due to the unavailability of tissue samples for the majority of patients, we could not investigate whether such a predictive value is maintained on subsequent biopsies. In this context, other circulating biomarkers, such as PHI and *PCA3*, assessed on baseline samples improved the accuracy of predicting reclassification at the first surveillance biopsy but did not provide further value over clinical variables on subsequent biopsies [8,9].

An AS program’s success is critically dependent on selecting patients who are predicted to have clinically insignificant cancer. Methods to improve selection tools are an active research area. Many researchers found that some clinicopathological features (PSA density, number of positive cores at diagnostic biopsy, T-stage) were associated with upgrading in AS [30]. However, the discriminative power of these models was too modest to allow their use as a refined tool for patient selection. For this reason, researchers started to investigate both imaging and biological biomarkers [31]. The miRNA-based signature presented here explores the possibility to better define aggressive vs. indolent diseases by using a blood biomarker. In terms of AUC value, our signature was comparable to that observed with the three-miRNA score proposed by Liu et al. [25] as well as with PHI [8] and *PCA3* [9] in the AS setting.

Coupling of our three-miRNA signature with selected clinicopathological variables, however, led to a statistically significant, although modest (6%), improvement in the discrimination of aggressive cancer, underlining possible complementary information coming from the miRNome. PSA density (<0.15 ng/mL) and the number of positive cores (≤2) at diagnosis are already used in international guidelines to discriminate very low-risk vs. low-risk patients [32]. Some AS protocols also use this information for risk assessment at enrollment (some others to schedule a different follow-up).

The addition of a biomarker that is biopsy-independent, as is the miRNA signature, could help to refine the classification of patients into subgroups, such as those having a “decreased risk” of misclassification at diagnosis (i.e., favorable PSA density, a limited number of positive cores and favorable miRNome) and those having an “increased risk” of misclassification (i.e., high PSA density, increased number of positive cores and high-risk miRNome). This improved knowledge of the single patient risk could help in counseling patients about treatment choice and personalized monitoring intensity during AS. To more precisely define its possible clinical utility for risk estimation, the three-miRNA signature should be investigated in contemporary AS protocols together with mpMRI, which was not routinely available in the great majority of patients at the time of this study.

Information concerning the biological functions in PCa of miRNAs included in the signature is only available for *miR-199a-5p*. Consistent with the downregulation observed in plasma samples from upgrading patients, it was reported that *miR-199a-5p* is under-expressed in PCa clinical specimens and acts as an oncosuppressor, since its reconstitution in PCa models decreased cell proliferation, motility and tumor angiogenesis and increased apoptosis by directly targeting HIF-1α [33]. As regards the other two miRNAs of the signature, *miR-511-5p* was reported tofunction as a tumor suppressor in colorectal cancer by directly targeting GPR116 [34], while *miR-598-3p* was found to be downregulated in serum samples from breast cancer patients compared to healthy individuals [35].

Overall, we identified and validated a plasma miRNA signature significantly associated with disease upgrading at the first repeated biopsy in three mono-institutional independent cohorts of AS patients. When combined with selected clinicopathological criteria, the miRNA signature enhanced their predictive value. However, it should be considered that any formal evaluation of the clinical utility of the signature depends on cutoff selection, which needs to be validated in *ad hoc* studies.

As the main limitation of the study is the absence of mpMRI data, the clinical utility of the signature as a novel biomarker to improve patient selection needs to be further validated in other cohorts of AS patients together with mpMRI.

## 5. Conclusions

We trained, tested and validated a plasma miRNA signature significantly associated with disease upgrading at the first repeated biopsy in three mono-institutional independent cohorts of AS patients. When combined with clinicopathological parameters, the miRNA signature enhanced their predictive value. The clinical utility of the signature as a novel biomarker to improve patient selection should be further validated in contemporary AS protocols together with mpMRI.

## Figures and Tables

**Table 1:** Clinicopathological characteristics of patients in the three AS cohorts.

	TRS ^a^		TES ^a^		VAS ^a^	
	*n* = 121		*n* = 111		*n* = 127	
Variable (at Diagnosis)	Median	IQR ^b^	Median	IQR ^b^	Median	IQR ^b^
Age (years)	64	59–70	62	58–66	63.4	58.1–69.2
PSA (ng/mL)	5.36	4.27–6.30	5.89	4.8–7.1	5.9	4.83–7.44
Prostate volume (cm^3^)	44	36–58	46	35–61	48	37–63
PSA density (ng/mL/cm^3^)	0.12	0.08–0.15	0.11	0.08–0.16	0.12	0.09–0.17
Total biopsy cores (*n*)	12	10–16	14	12–16	12	12–16
Max PCa length (%)	10	5–20	5	5–20	10	5–20.5
Positive cores (*n*, %)						
<10	62 (51.24%)		64 (57.66%)		52 (40.94%)	
≥10	59 (48.76%)		47 (42.34%)		75 (59.06%)	
Positive cores (*n*, %)						
≤1	85 (70.25%)		71 (63.96%)		57 (44.88%)	
>1	36 (29.75%)		40 (36.04 %)		70 (55.12%)	
Gleason Pattern Score/Prognostic Grade Group (*n*, %)						
GPS = /PGG1	121 (100%)		111 (100%)		127 (100%)	
Clinical Stage (*n*, %)						
T1b	-		-		1 (0.79%)	
T1c	113 (93.39%)		106 (95.5%)		122 (96.06%)	
T2a	8 (6.61%)		5 (4.5%)		4 (3.15%)	

^a^ TRS = training set; TES = testing set; VAS = validation set; ^b^ IQR = interquartile range.

**Table 2:** List of 17 hemolysis-free candidate miRNAs from univariate analysis on training set.

miRNA	OR (95% CI)	*p*-Value	
*miR-122-5p*	1.412 (1.007;1.979)	0.046	*
*miR-1255b-5p*	1.590 (1.000;2.528)	0.045	†
*miR-128a-3p*	0.380 (0.153;0.944)	0.037	*
*miR-142-5p*	0.353 (0.138;0.903)	0.030	*
*miR-181c-5p*	0.634 (0.439;0.916)	0.015	*
*miR-199a-5p*	0.663 (0.402;1.095)	0.043	†
*miR-204-5p*	1.428 (0.929;2.195)	0.035	**
*miR-27b-3p*	0.421 (0.219;0.809)	0.010	*
*miR-324-5p*	0.471 (0.266;0.836)	0.010	*
*miR-330-3p*	0.387 (0.172;0.873)	0.022	*
*miR-337-3p*	0.694 (0.429;1.123)	0.048	†
*miR-361-5p*	0.479 (0.236;0.973)	0.042	*
*miR-422a*	1.451 (0.980;2.147)	0.039	†
*miR-502-5p*	1.497 (0.991;2.262)	0.049	†
*miR-511-5p*	1.568 (0.976;2.518)	0.044	†
*miR-572*	1.720 (1.034;2.860)	0.037	*
*miR-598-3p*	0.395 (0.151;1.036)	0.048	†

† Nonparametric Kruskal–Wallis *p*-value; * odds ratio (OR) *p*-value from univariate logistic regression model; ** AUC *p*-value from univariate logistic regression model.

**Table 3:** Results from the univariate and multivariate logistic models on the overall cohort (clinico-pathological variables).

	Univariate Analysis				Multivariate Analysis	
Variables	UPG *	IND **	OR ^†^	95% CI	OR	95% CI
Age (years)	65	294	1.074	1.029–1.120	1.081	1.033–1.132
PSA density (ng/mL/cm^3^)	65	294	2.599	1.392–4.853	2.677	1.418–5.053
Prostate volume (cm^3^)	65	294	0.469	0.264–0.833	–	
Positive cores (*n*)	65	294	1.923	1.119–3.306	–	
Positive cores (%)	65	294	2.036	1.166–3.556	–	
Max PCa length (%)	62	285	1.024	1.009–1.040	1.024	1.008–1.040

Age: age at biopsy(continuous scale); PSA density: PSA/Volume (continuous logarithmic scale); Volume: prostatic volume (dichotomized as <50cc vs. ≥50cc, according to Marenghi et al. [14]); Positive cores (*n*): number of positive cores at diagnostic biopsy (dichotomized as ≤1 vs. >1); Positive cores (%): % positive cores at diagnostic biopsy (dichotomized as <10% vs. ≥10%); Max PCa length (%): maximum length of prostate cancer in positive core. * Number of upgrading patients from all the cohorts; ** number of indolent patients from all the cohorts; ^†^ odds ratio.

## Data Availability

The datasets used and/or analyzed during the current study are available from the corresponding author upon reasonable request.

## Supplementary Materials

The following are available online at https://www.mdpi.com/article/10.3390/cancers13102433/s1, Figure S1: Heatmap depicting the level of significance of the candidate miRNAs arising from the different univariate analysis in the TRS, Figure S2: Results obtained by considering all the available variables in the clinicopathological model, Table S1: Inclusion criteria, follow-up schedule and indications for discontinuation of Active Surveillance protocols at Istituto Nazionale Tumori, Milan, Table S2: Pairwise relationships of the available clinicopathological variables.
